# Are there avoidable causes of early revision total knee arthroplasty?

**DOI:** 10.1186/s43019-022-00157-z

**Published:** 2022-06-18

**Authors:** Mackenzie A. Roof, Jason B. Kreinces, Ran Schwarzkopf, Joshua C. Rozell, Vinay K. Aggarwal

**Affiliations:** Department of Orthopedic Surgery, NYU Langone Orthopedic Hospital, NYU Langone Health, 462 First Ave (BHC), CD Bldg Room 4-85, New York, NY USA

**Keywords:** Total knee arthroplasty, Revision total knee arthroplasty, Unavoidable, Avoidable, Complications

## Abstract

**Introduction:**

Surgeons can improve their practice by understanding potentially avoidable reasons for early revision total knee arthroplasty (rTKA). The purpose of this study is to describe potentially avoidable indications for rTKA within 5 years of the index procedure.

**Methods:**

This retrospective observational analysis utilized the rTKA database at a large, academic orthopedic specialty hospital to identify 167 consecutive rTKA from 2014 to 2019 performed within 5 years after primary TKA (pTKA). Two fellowship-trained arthroplasty surgeons reviewed the cases to classify them as potentially avoidable or unavoidable. Unavoidable indications for rTKA included infection/wound complication, arthrofibrosis, instability due to underlying collagen disease or significant weight loss, and any traumatic event leading to pTKA failure. Potentially avoidable indications included atraumatic instability, intraoperative fracture, metal allergy, and atraumatic patellar instability or maltracking. Aseptic loosening was considered avoidable if it occurred in the presence of component malpositioning or poor cementation technique.

**Results:**

There were 112 (67.1%) unavoidable cases and 55 avoidable cases (32.9%). Of the unavoidable cases, there were 68 rTKA for infection or wound complications (60.7%), 23 for arthrofibrosis (20.5%), 6 for instability (5.4%), 6 for postoperative fracture (5.4%), 6 for aseptic loosening (5.4%), and 3 for extensor mechanism pathology following trauma (2.6%). Of the potentially avoidable rTKA, 24 were for aseptic loosening (43.7%), 23 for atraumatic instability (41.8%), 6 for atraumatic extensor mechanism pathology (10.9%), 1 for nickel allergy (1.8%), and 1 for intraoperative fracture (1.8%).

**Conclusion:**

These findings indicate that over 30% of early rTKA are potentially avoidable. Interventions should focus on addressing potentially avoidable causes such as short-term aseptic loosening and instability to reduce the need for costly and resource-intensive rTKA.

*Level of evidence* III, retrospective observational analysis

## Introduction

Total knee arthroplasty (TKA) is a well-established treatment for end-stage arthritis with excellent functional outcomes and survivorship rates exceeding 95% at 5-year follow-up [1–3]. Additionally, the majority of patient-reported outcomes following TKA fulfill or exceed patient expected satisfaction [4]. Over the past decade in the USA, there has been a large increase in the number of primary (pTKA) and revision TKA (rTKA) performed, with approximately 5.4 rTKA per 100,000 persons [5]. A substantial increase in demand for these procedures is expected by 2030, including a larger contribution from patients younger than 65 years old [5, 6]. The projected increase in demand requires the deployment of appropriate preventative and diagnostic measures to minimize postoperative complications.

Despite the success of pTKA, data from a US Nationwide Impatient Sample (NIS) study conducted by Bozic et al. in 2010 demonstrated that the number of rTKA increased from 48,260 in 2006 to 67,534 in 2010 [7]. This represented a 39% increase in revision procedures, which is likely to continue given the substantial projected rise in pTKA cases by 2030 [7]. rTKA procedures require more hospital resources and are often associated with greater hospital cost, longer length of stay, and longer operative time [3, 8, 9]. As a result, rTKA impose a serious economic burden on the US healthcare system, with projections estimating rTKA cost to rise to US $13 billion annually by 2030 [5, 10]. In addition to economic challenges, rTKA procedures have worse survivorship. Compared with the 97.2% 5-year survivorship rate of pTKA, rTKA have an 87.4% 5-year survivorship rate [3]. It is crucial for orthopedic surgeons to critically evaluate their pTKA cases to minimize avoidable rTKA to mitigate potential economic and health-related complications.

Infection and implant loosening are the two most common indications for rTKA [11]. This finding is consistent across the literature, highlighting the importance of identifying ways to address these indications for rTKA [3, 7, 8, 11, 12]. Avoidable revisions indications are defined as those that result from an insufficiency of care by the medical provider as determined by the provider’s peers [13]. Potential avoidable indications for revision have been investigated in total hip arthroplasty (THA), but from our literature review, no studies have been published looking at potential avoidable indications for rTKA [14].

Surgeons can improve their surgical practice by understanding potentially avoidable reasons for rTKA. The purpose of this study was to describe potentially avoidable indications for rTKA within 5 years of the index procedure. We hypothesized that a significant portion of rTKA may be potentially avoidable and that indications for rTKA would differ between avoidable and unavoidable cases.

## Methods

### Data collection

This retrospective observational analysis examined the rTKA database at a large academic orthopedic specialty hospital. After receiving approval from our Institutional Review Board (IRB), 1171 consecutive rTKA performed between June 2014 and June 2019 were reviewed. Of these, 946 patients either did not have their index pTKA performed at our institution or had their pTKA performed greater than 5 years before their rTKA. We chose to solely look at rTKA performed within 5 years of pTKA because these early rTKA are more likely to be related to the original surgical technique and are therefore the most burdensome to the healthcare environment, especially if potentially avoidable. Additionally, we chose to only include patients who had their pTKA performed at our institution to ensure that all necessary operative and radiographic information would be available. This left 225 patients for review. One patient who received a hinged knee construct at pTKA was excluded, as well as 57 patients who did not have radiographic imaging available within 1 year post-pTKA. Therefore, a total of 167 patients who underwent rTKA between June 2014 and June 2019 were ultimately included.

Two fellowship-trained arthroplasty surgeons independently reviewed the post-pTKA radiographs and medical records associated with each case and classified the rTKA into one of two possible categories based on surgical technique. Unavoidable cases meant that the patient was optimally managed, whereas potentially avoidable cases meant that the patient’s care could have been better optimized. Based on chart review of the cases, all patients were medically preoptimized and managed appropriately with no untoward medical complications in the postoperative period. This methodology was adapted from a 2019 study by Novikov et al. looking at potentially avoidable causes of rTHA [14, 15].

In the present study, unavoidable indications for rTKA included infection/wound complication, arthrofibrosis, instability due to underlying collagen disease or significant postoperative weight loss, and any traumatic event leading to failure of a previously well-functioning TKA, such as extensor mechanism rupture or postoperative periprosthetic fracture. Instability in patients with a properly balanced primary TKA and appropriate component positioning was attributed to ligamentous laxity and classified as unavoidable. Patient falls and postoperative stiffness due to lack of patient engagement were deemed unavoidable by the surgeons. Infection was determined utilizing the 2018 Musculoskeletal Infection Society (MSIS) criteria [16]. Patients with an MSIS score greater than or equal to 6 were determined to be revised for periprosthetic joint infection (PJI). Patients with an MSIS score less than 6 were determined to be revised for wound complication. At our institution, we utilize a Perioperative Surgical Home (POSH) protocol to optimize patients in the preoperative period to minimize the burden of infectious complications [17]. Through this protocol, we utilize a co-management strategy with medicine hospitalists dedicated to the care of postoperative patients with specific regards to glucose control and anticoagulation, which involves standardized aspirin dosing to avoid excessive bleeding. Therefore, patients in this cohort who developed PJI or wound complications did so despite preoperative and postoperative optimization, which is standard of care at our institution. The use of intraoperative tourniquet and technology in study patients was based on surgeon preference and representative of the overall practices for pTKA at our institution. All pTKA were performed using cemented technique.

Potentially avoidable indications included instability for atraumatic reasons, intraoperative fracture, metal allergy, and atraumatic patellar instability or maltracking. Although intraoperative fracture is a known risk of rTKA, it is not a standard complication, and we believe that it can be avoided with appropriate preoperative planning and intraoperative technique. Aseptic loosening was considered avoidable if it occurred in the presence of component malpositioning or poor cementation technique, which were identified by radiograph and chart review. While metal allergy, even if present, may not affect all patients with equal clinical significance, the preoperative process at our institution includes thorough questioning of all allergies, including metal. As many patients with a positive patch test may not have an inflammatory response after the implant is placed, we do not routinely send patients who are sensitive to metal for patch testing; rather, we use hypoallergenic/non-cobalt chrome-containing implants to avoid this issue.

Baseline demographic information including sex, age, and body mass index (BMI) were collected. Further review of each patient examined perioperative details, including indications for pTKA, use of intraoperative assistive technology, type of primary construct (posterior stabilized versus cruciate retaining), polyethylene size, and anesthesia type. Postoperative details including time to most recent follow-up, re-rTKA, and time to rTKA were also recorded. The standard of care at our institution is that all patients have full passive ROM when leaving the operative room, which was documented in all operative reports.

**Fig. 1:**
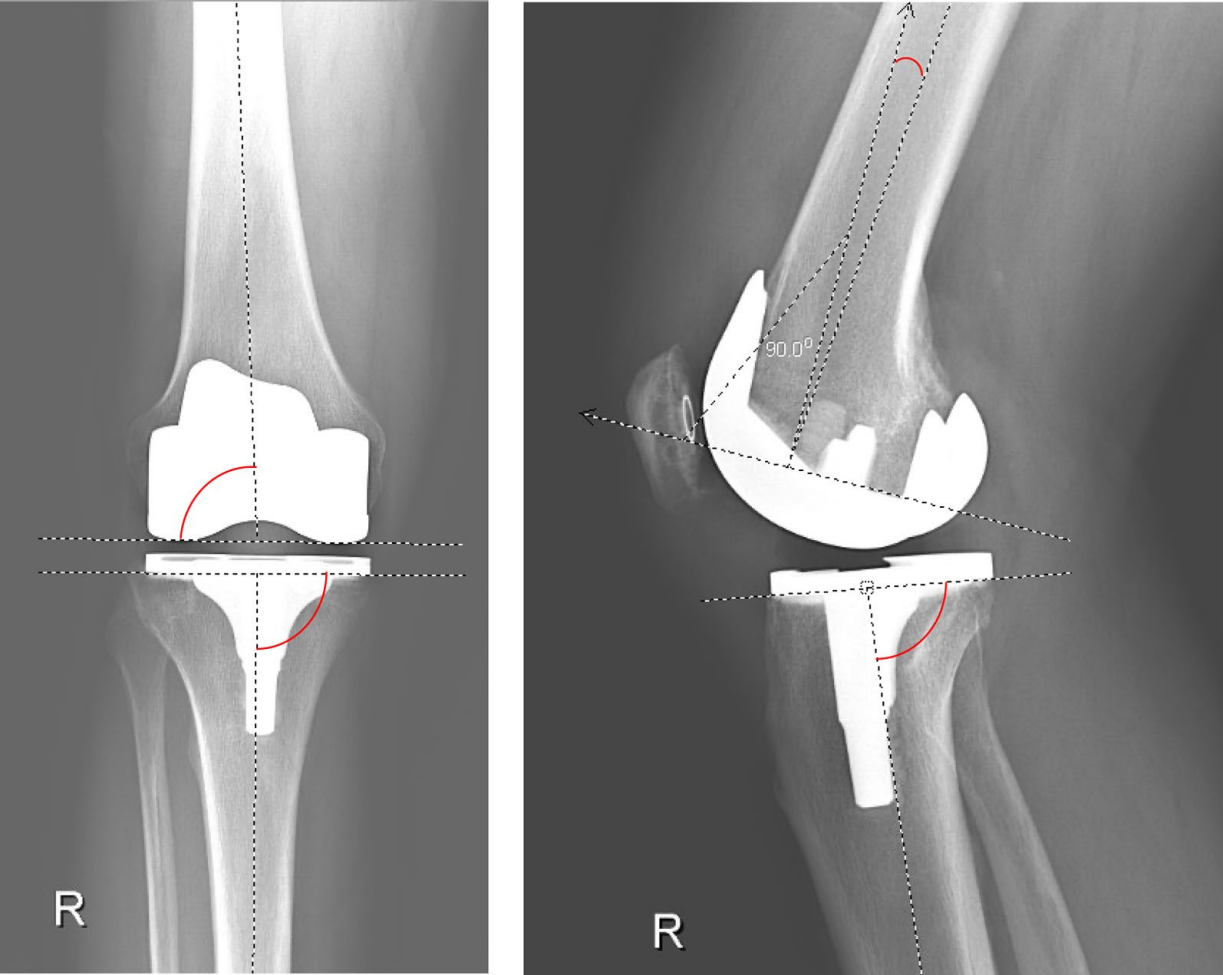
Radiograph showing the measurement of four component alignment angles. *LDFA* lateral distal femoral angle, *MPTA* medial proximal tibial angle (MPTA), *FFA* femoral flexion angle, *TS* tibial slope

Radiographic parameters, including the lateral distal femoral angle (LDFA), medial proximal tibial angle (MPTA), femoral flexion angle (FFA), and tibial slope (TS), were measured using a digital measurement tool provided by the hospital picture archiving and communications system (PACS). The LDFA and MPTA were both measured on standardized anterior–posterior (AP) knee radiographs for all patients who underwent rTKA (Fig. 1). The FFA and TS were both measured on standardized lateral knee radiographs for all patients who underwent rTKA (Fig. 1). All radiographic images were taken prior to rTKA within 1 year of the index procedure. Cementation technique was evaluated using a modified version of the procedures outlined by Meneghini et al. [18] and Ozkunt et al. [19]. We did not manually measure cement penetration as there has been no study definitively showing that there is an optimal depth of cementation for rTKA.

### Statistical analysis

Proportions were utilized to compare the rates of potentially avoidable and unavoidable rTKA. Chi-squared analysis was utilized to analyze categorical data. Continuous data were analyzed using Student’s *t*-test. IBM SPSS Statistics version 25 (Armonk, NY) was utilized for the statistical analyses. *p*-Values less than 0.05 were considered statistically significant.

## Results

### Patient demographics

A total of 55 rTKA (32.9%) were classified as potentially avoidable, and 112 rTKA (67.1%) were classified as unavoidable. The baseline demographics at the time of pTKA were similar between the cohorts (Table 1). Patients in the potentially avoidable group were revised at a significantly later time than the patients in the unavoidable group (17.89 versus 10.47 months; *p* < 0.001; Table 1). Patients in the potentially avoidable group also had significantly shorter post-rTKA follow-up than patients in the unavoidable group (16.70 versus 23.87 months; *p* = 0.004; Table 1).

**Table 1:** Patient demographics at time of primary TKA

Demographic	Potentially avoidable (*n* = 55)	Unavoidable (*n* = 112)	*p*-Value
Patient age (mean years ± SD)^b^	62.09 ± 7.86	60.72 ± 10.38	0.345
Sex, *n* (%)^a^			
Female	39 (70.9)	67 (59.8)	0.176
Male	16 (29.1)	45 (40.2)	
Race, *n* (%)^a^			
White	26 (47.3)	60 (53.6)	0.220
Black	14 (25.5)	27 (24.1)	
Asian	2 (3.6)	0 (0.0)	
Other	13 (23.6)	25 (22.3)	
Smoking status, *n* (%)^a^			
Never	30 (54.5)	54 (48.2)	0.742
Former	20 (36.4)	46 (41.1)	
Current	5 (9.1)	12 (10.70	
Laterality, *n* (%)^a^			
Right	29 (52.7)	56 (50.0)	0.745
Left	26 (47.3)	56 (50.0)	
Diabetes mellitus, *n* (%)^a^	17 (30.9)	34 (30.4)	1.000
Inflammatory arthritis, *n* (%)^a^	6 (10.9)	11 (9.8)	0.792
BMI (mean kg/m^2^ ± SD)^b^	33.85 ± 7.33	34.82 ± 7.24	0.421
ASA score, *n* (%)^a^			
1	0 (0.0)	2 (1.8)	0.134
2	36 (65.5)	54 (48.2)	
3	19 (34.5)	54 (48.2)	
4	0 (0.0)	2 (1.8)	
Indication for TKA, *n* (%)^a^			
Osteoarthritis	53 (96.4)	102 (91.1)	0.217
Posttraumatic arthritis	0 (0.0)	6 (5.4)	
Inflammatory arthritis	2 (3.6)	4 (3.6)	
Prior ipsilateral knee surgery, *n* (%)^a^	22 (40.0)	45 (40.2)	1.000
Time to rTKA (mean months ± SD)^b^	17.89 ± 13.47	10.47 ± 11.88	< 0.001**
Mean follow-up since rTKA (mean months ± SD)^b^	16.70 ± 13.78	23.88 ± 16.62	0.004**

*TKA* total knee arthroplasty, *SD* standard deviation, *BMI* body mass index, *ASA* American Society of Anesthesiologists

^a^Chi-square/Fisher’s exact test

^b^Student’s *t*-test

*p < 0.05; **p < 0.01

### Surgical factors

Perioperative characteristics including anesthesia type, construct, polyethylene liner thickness, and technological assistance were statistically similar in the potentially avoidable and unavoidable rTKA groups (Table 2). Tourniquet time and the duration of surgery were also similar (Table 2).

**Table 2:** Primary TKA information

Characteristic	Potentially avoidable (*n* = 55)	Unavoidable (*n* = 112)	*p*-Value
Anesthesia, *n* (%)^a^			
General	5 (9.1)	17 (15.2)	0.336
Regional	50 (90.9)	95 (84.8)	
Construct, *n* (%)^a^			
Posterior stabilized	37 (67.3)	78 (69.6)	0.859
Cruciate retaining	18 (32.7)	31 (27.7)	0.588
Liner thickness, *n* (%)^a^			
< 9 mm	2 (3.6)	7 (6.3)	0.685
9–14 mm	46 (83.6)	94 (83.9)	
> 14 mm	7 (12.7)	11 (9.8)	
Technology, *n* (%)^a^			
Navigation	3 (5.5)	6 (5.4)	1.000
Robotics	0 (0.0)	3 (2.7)	0.552
OrthoSensor	5 (9.1)	10 (8.9)	1.000
Tourniquet use, *n* (%)^a^	52 (94.5)	102 (91.1)	0.552
Tourniquet time (mean minutes ± SD)^b^	86.80 ± 28.06	93.28 ± 32.18	0.205
Surgical time (mean minutes ± SD)^b^	70.04 ± 22.12	75.52 ± 22.98	0.171

*TKA* total knee arthroplasty, *SD* standard deviation

^a^Chi-square/Fisher’s exact test

^b^Student’s *t*-test

*p < 0.05; **p < 0.01

Most pTKA in the potentially avoidable group were performed under regional anesthesia (50; 90.9%), using a posterior stabilized femoral implant (37; 67.3%), had a polyethylene liner thickness between 9 and 14 mm (46; 83.6%), and used a tourniquet (52; 94.5%). Intraoperative assistive technology was used in a total of eight potentially avoidable cases, with OrthoSensor (OrthoSensor Inc., Dania Beach, Florida) being the most used technology (5; 9.1%).

Most pTKA in the unavoidable group were also performed under regional anesthesia (95; 84.8%), using a posterior stabilized femoral implant (78; 69.6%), had a polyethylene liner thickness between 9 and 14 mm (94; 83.9%), and used a tourniquet (102; 91.1%). Intraoperative assistance technology was used in 19 of the unavoidable cases, with OrthoSensor being the most used technology (10; 8.9%).

### Post-primary TKA outcomes

There were significantly more 90-day readmissions in the unavoidable group (55, 49.1% versus 7, 12.7%, *p* < 0.001; Table 3). In the potentially avoidable group, the mean hospital length of stay following pTKA was 2.62 days (SD, 1.35 days). Most patients were discharged home following the pTKA (47; 85.5%). Seven patients were readmitted within 90 days (10.9%), and six patients had a non-revision surgery (manipulation under anesthesia, arthroscopy, irrigation and debridement, extensor mechanism repair, arthrotomy with lateral release) following pTKA but before rTKA (10.9%). In the unavoidable group, the mean hospital length of stay following pTKA was 3.01 days (SD, 1.56 days). Most patients were also discharged home following the pTKA (91; 81.3%). Fifty-five patients were readmitted within 90 days (49.1%), and 23 patients had a non-revision surgery following pTKA but before rTKA (20.5%).

**Table 3:** Post-primary TKA outcomes

Characteristic	Potentially avoidable (*n* = 55)	Unavoidable (*n* = 112)	*p*-Value
Hospital length of stay (mean days ± SD)^b^	2.62 ± 1.35	3.01 ± 1.56	0.114
Discharge disposition, *n* (%)^a^			
Home	47 (85.5)	91 (81.3)	0.749
Skilled nursing facility	5 (9.1)	15 (13.4)	
Acute rehabilitation facility	3 (5.5)	5 (4.5)	
Short-term general hospital	0 (0.00)	1 (0.9)	
90-Day readmissions, *n* (%)^a^	7 (12.7)	55 (49.1)	< 0.001**
Non-revision surgery between primary and revision, *n* (%)^a^	6 (10.9)	23 (20.5)	0.135

*TKA* total knee arthroplasty, *SD* standard deviation

^a^Chi-square/Fisher’s exact test

^b^Student’s *t*-test

*p < 0.05; **p < 0.01

### Radiographic evaluation

Radiographic analysis of mean component alignment angles in the coronal and sagittal planes revealed no significant differences in the LDFA, MPTA, FFA, and TS between the potentially avoidable and unavoidable patient groups (Table 4).

**Table 4:** Post-primary radiographic measurements

Measurement	Potentially avoidable (*n* = 55)	Unavoidable (*n* = 112)	*p*-Value
Lateral distal femoral angle (mean degrees ± SD)	84.66 ± 2.53	85.11 ± 2.87	0.330
Medial proximal tibial angle (mean degrees ± SD)	88.945 ± 2.39	88.312 ± 2.58	0.128
Femoral flexion angle (mean degrees ± SD)	4.67 ± 3.44	4.48 ± 3.92	0.756
Tibial slope (mean degrees ± SD)	85.44 ± 2.99	86.23 ± 2.89	0.101

Student’s *t*-test

*p < 0.05; **p < 0.01

### Indications for revision

The potentially avoidable indications for rTKA were aseptic loosening (24; 43.7%), instability (23; 41.8%), extensor mechanism issues (6; 10.9%), intraoperative fracture (1; 1.8%), and nickel allergy (1; 1.8%) (Table 5).

**Table 5:** Revision indications

Indication	*N* (%)
Avoidable indications	
Aseptic loosening	24 (43.7)
Instability	23 (41.8)
Intraoperative fracture	1 (1.8)
Extensor mechanism issues	6 (10.9)
Nickel allergy	1 (1.8)
Unavoidable indications	
Arthrofibrosis	23 (20.5)
Aseptic loosening	6 (5.4)
Instability	6 (5.4)
Infection/wound complication	68 (60.7)
Postoperative fracture	6 (5.4)
Extensor mechanism issue	3 (2.6)

The indications for unavoidable rTKA were infection/wound complication (68; 60.7%), arthrofibrosis (23; 20.5%), aseptic loosening (6; 5.4%), instability (6: 5.4%), postoperative fracture (6; 5.4%), and extensor mechanism issue (3; 2.6%) (Table 5). Of the infection/wound complication group, 58 (85.3%) were revised for PJI based on the MSIS criteria and 10 (14.7%) were revised for wound complications.

### Aseptic loosening

Subgroup analysis was conducted comparing the avoidable and unavoidable cases revised for aseptic loosening. This analysis did not show a significant difference in any angle measurements (LDFA *p* = 0.765; MPTA *p* = 0.323; FFA *p* = 0.272; TS *p* = 0.741) between the potentially avoidable and unavoidable groups revised for aseptic loosening. Of the 30 patients revised for aseptic loosening, 24 were classified as avoidable rTKA. Of these 24 patients, 15 were classified as such due to poor cementation technique as evaluated on the first postoperative radiograph.

### Post-revision TKA outcomes

Post-rTKA outcomes including hospital length of stay, discharge disposition, 90-day readmissions, and re-revision procedures were statistically different between the two groups (Table 6). Mean hospital length of stay was significantly shorter in the potentially avoidable group than the unavoidable group (2.36 versus 5.97 days; *p* < 0.001; Table 6). In the potentially avoidable group, significantly more patients were discharged home compared with the unavoidable group (51; 92.7% versus 82; 73.2%; *p* = 0.010; Table 6). The potentially avoidable group also had a significantly lower number of 90-day readmissions compared with the unavoidable group (3; 5.5% versus 25; 23.2%; *p* = 0.004; Table 6). The unavoidable group had a higher number of re-revisions compared with the potentially avoidable group (6; 10.9% versus 60; 53.6% versus; *p* < 0.001; Tables 6, 7, 8).

**Table 6:** Post-revision TKA outcomes

Characteristic	Potentially avoidable (*n* = 55)	Unavoidable (*n* = 112)	*p*-Value
Hospital length of stay (mean days ± SD)^b^	2.36 (1.38)	5.97 (5.44)	< 0.001**
Discharge disposition, *n* (%)^a^			
Home	51 (92.7)	82 (73.2)	0.010*
Skilled nursing facility	4 (7.3)	24 (21.4)	
Acute rehabilitation facility	9 (0.0)	6 (5.4)	
90-Day readmissions, *n* (%)^a^	3 (5.5)	26 (23.2)	0.004**
Any re-revision, *n* (%)^a^	6 (10.9)	60 (53.6)	< 0.001**

Re-revision: components were exchanged

^a^Chi-square/Fisher’s exact test

^b^Student’s *t*-test

*p < 0.05; **p < 0.01

**Table 7:** Etiology of instability as indication for revision

Potentially avoidable (*n* = 55)	*N*	Unavoidable (*n* = 112)	*N*
Improper component sizing	7	Ehlers–Danlos	1
Component malpositioning	2	Weight loss	1
Polyethylene liner loosening	1	Traumatic ligament damage	4
		Tibial plateau fracture, failure into varus	1

**Table 8:** Indications for post-primary 90-day readmissions

Potentially avoidable (*n* = 7)	*N*	Unavoidable (*n* = 55)	*N*
MUA/arthroscopy	1	Infection	43
Abdominoplasty	1	MUA/arthroscopy	5
Component shifting	2	Fracture	3
Extensor mechanism issues	2	Contralateral TKA	2
Arthrotomy, lateral release	1	Extensor mechanism issues	1
		Arthrotomy, lateral release	1

Indications for post-revision 90-day readmissions			
Potentially avoidable (*n* = 3)	*N*	Unavoidable (*n* = 26)	*N*
Wound repair	1	Infection	20
Colonoscopy	1	MUA	2
Cirrhosis	1	Influenza infection	1
		Gastrointestinal bleed	1
		Fever	1
		Contralateral TKA	1

Indications for re-revision			
Potentially avoidable (*n* = 6)	*N*	Unavoidable (*n* = 60)	*N*
Aseptic loosening	4	Infection	47
Instability	1	Extensor mechanism issues	2
Fracture	1	Arthrofibrosis	4
		Instability/dislocation	3
		Aseptic loosening	3
		Pain	1

## Discussion

Despite the utility of TKA, rTKA procedures are more technically demanding and confer increased cost and potentially serious health complications for patients. Many studies indicate that infection, implant loosening, and instability are the leading indications for rTKA [3, 7–9, 11, 12]. While often discussed, no data are available on whether rTKA procedures might have been avoidable. Our study is the first to consider whether rTKA might be avoidable and showed that a large proportion of rTKA (32.9%) are potentially avoidable. Our study is also the first to evaluate indications for avoidable rTKA and determined that aseptic loosening (43.7%) and instability (41.8%) were responsible for the majority of the potentially avoidable rTKA. The methodology of the pTKA included in this study were similar between the two cohorts and representative of the overall pTKA methodology at our institution with respect to implants, tourniquet use, and technology assistance.

Identification of rTKA as unavoidable or potentially avoidable is an important first step in reducing the burden of these costly procedures. While risk of PJI is potentially minimized by optimizing patient risk factors preoperatively, we felt that infection is difficult to directly avoid intraoperatively as we used the same aseptic technique for all pTKA at our institution. Furthermore, all patients in this study were optimized using the POSH protocol previously published by our institution [17]. Postoperative periprosthetic fracture or extensor mechanism rupture from traumatic events leading to failure are also outside of the surgeon’s control as they are directly related to patient activity in the postoperative period. Arthrofibrosis is a poorly understood complication following TKA and is likely related to an exaggerated inflammatory response that is difficult to predict [20]. While collagen disease can be diagnosed in the preoperative period, it is challenging to predict how the patient’s ligamentous structures will adapt to TKA implantation, especially in the setting of postoperative weight loss (Table 7).

Accurate implant positioning is an important surgeon-controlled variable that influences pTKA functional outcomes, implant stability, and implant survivorship [21]. Numerous studies have shown that successful long-term TKA outcomes depend on proper component positioning and femoral and tibial alignment [21–23]. With the development of advanced technology, suboptimal component positioning may be the easiest target to reduce the need for rTKA. Computer-navigated and robotic TKA have recently gained momentum as methods for improving the accuracy of implant positioning [21]. Previous authors have highlighted that robotic TKA results in greater accuracy in implant positioning and limb alignment compared with conventional TKA [21, 23–27]. Bellemans et al. [27] reported that robotic TKA resulted in femoral and tibial implant positioning within 1° of planned positions in the coronal, sagittal, and axial planes. Similarly, Cheng et al. [28] reported that computer-assisted TKA cases showed statistically significant reductions in the number of patients with malalignment of the femoral and tibial components. These technologies may reduce outliers; however, their effect on rates of aseptic loosening or instability resulting from incorrect component positioning or sizing has yet to be definitively identified.

To this end, we evaluated four angles on the post-pTKA radiographs for each patient, including the LDFA, MPTA, FFA, and TS. We did not see a difference in average radiographic parameters between unavoidable and potentially avoidable study groups overall. This is likely due to our small cohort, as only one of the four radiographic parameters may be affected in a patient, diluting the mean angle measurements available for comparison between the two groups. Furthermore, although we classified significantly more patients with aseptic loosening in the avoidable group as compared with the unavoidable group (43.7% versus 5.4%), more than half (15/24, 62.5%) of the avoidable aseptic loosening patients were classified as such due to poor cementation of components. To avoid the complication of poor cementation at our institution, we emphasize either the use of tourniquet during cementing or the use of high-flow gas bone preparation to decrease fat and blood in the bones for optimal cement interdigitation. We also apply cement to bone with hand pressurization and cement to the implant.

To further investigate the role of surgical precision in the development of aseptic loosening necessitating rTKA, a subgroup radiographic analysis between the potentially avoidable and unavoidable cases of aseptic loosening was conducted. This analysis did not show a significant difference in any angle measurements between the potentially avoidable and unavoidable groups revised for aseptic loosening. Therefore, while careful evaluation of component positioning and sizing is necessary to avoid catastrophic implant failure, our results show that the etiology of aseptic loosening is likely multifactorial and may not be solely predictable by objective parameters such as malalignment. As such, further research investigating potential direct causes of aseptic loosening are being undertaken at our institution.

While the results of our study are encouraging in that many rTKA procedures may be avoidable, a large proportion of rTKA were determined to be unavoidable. Expectantly, this study demonstrated that patients who underwent unavoidable rTKA had worse post-pTKA and post-rTKA outcomes than patients who underwent potentially avoidable rTKA surgery. This finding makes sense as the majority of patients who underwent unavoidable rTKA were revised for infection or wound complication (60.7%). The urgency to intervene on these two indications may explain the finding that patients in the potentially avoidable group were revised at a significantly later time than the patients in the unavoidable group. Revision for aseptic loosening or instability was based on failure of nonoperative management in the first two postoperative years, including use of medical management, physical therapy, and serial radiographs. If patients had no improvement and obvious signs of loosening or ligamentous laxity on examination, they proceeded to a revision operation.

Infections following TKA are a devastating complication, often necessitating multiple surgeries involving irrigation and debridement of the joint, placement and removal of antibiotic spacers, and possible limb amputation in refractory cases. This may explain the higher re-revision rate in this cohort. Additionally, these patients are often admitted to the hospital for a prolonged period for intravenous antibiotic treatment, likely contributing to the longer hospital stays.

Over the past several decades, identification of risk factors for infection as well as ways to prevent this complication have been under investigation. Although we classified infection as an unavoidable cause of rTKA, several patient-associated factors such as obesity, uncontrolled diabetes, smoking, and poor dental health are potentially modifiable risk factors for infection and rTKA. Kee et al. [29] determined that smoking and obesity are risk factors for revision arthroplasties of both the hip and knee for infection and aseptic loosening, and that infected rTKA patients were more likely to smoke and have poor dentition. Surgeons may be able to reduce rTKA for infection by proper patient evaluation and perioperative optimization with appropriate referrals to manage these risk factors prior to primary arthroplasty [29]. At our institution, we routinely screen for modifiable risk factors for PJI as published in our Perioperative Surgical Home (POSH) protocol [17]. Despite our efforts to minimize infection, the current study demonstrates that a significant proportion of rTKA are still due to infectious complications, including PJI and wound issues. This suggests that substantial work remains to understand these issues and develop protocols to reduce their incidence following TKA.

The current study emphasizes the need for surgeons to become familiar with modes of failure after pTKA and to be critical of their patient selection, surgical planning, and surgical technique. Our results suggest that approximately 30% of rTKA are potentially avoidable. In addition to the large costs associated with revisions, patients undergoing rTKA have a greater risk of complications and mortality [1, 3, 30].

### Limitations

This study has several limitations. First, due to its retrospective design and inclusion of only patients who had both their pTKA and rTKA performed at our institution, this study is at risk for selection bias and there may be missing data that limits our analysis. Additionally, differences in specificity and methodology of documentation between surgeons makes it challenging to further subclassify many of the indications, most notably with respect to instability, as there are a myriad of ways to document an instability examination. Second, although we sought to remain as objective as possible when evaluating the indication for rTKA, it is ultimately a subjective evaluation as there are no clear criteria against which to evaluate whether a rTKA was potentially avoidable or unavoidable. We attempted to mitigate this issue by having two fellowship-trained arthroplasty surgeons review each patient’s chart. Third, as indications for rTKA are not always mutually exclusive, it is possible that some patients had more than one underlying issue, which was not addressed in the analysis. Additionally, certain complications, such as metal hypersensitivity, have differing levels of clinical effects in different patients. Therefore, it is possible that unidentified reasons for indication are also present, contributing to TKA failure. Future studies should seek to establish criteria for avoidable and unavoidable rTKA, and larger studies should be undertaken to continue evaluating this important issue.

## Conclusions

A large proportion of rTKA are potentially avoidable. Careful evaluation for the indications for rTKA may enable surgeons to adapt their practice to address these issues and prevent future rTKA. The most common causes of avoidable rTKA were aseptic loosening and atraumatic instability, while unavoidable rTKA was seen most commonly in infection/wound complications. Femoral and tibial component positioning is an important area for surgeons to address to optimize accuracy of implant positioning. In addition to reduced health complications and mortality risk, reducing the incidence of potentially avoidable rTKA can greatly reduce the economic burden placed on the US healthcare system.

## Data Availability

The datasets generated during and/or analyzed during the current study are not publicly available due to ownership by NYU Langone Health but are available from the corresponding author on reasonable request.
